# Registration and Summation of Respiratory-Gated or Breath-Hold PET Images Based on Deformation Estimation of Lung from CT Image

**DOI:** 10.1155/2016/9713280

**Published:** 2016-12-19

**Authors:** Hideaki Haneishi, Masayuki Kanai, Yoshitaka Tamai, Atsushi Sakohira, Kazuyoshi Suga

**Affiliations:** ^1^Center for Frontier Medical Engineering, Chiba University, Chiba 263-8522, Japan; ^2^Graduate School of Engineering, Chiba University, Chiba 263-8522, Japan; ^3^St. Hill Hospital, Ube 755-0155, Japan

## Abstract

Lung motion due to respiration causes image degradation in medical imaging, especially in nuclear medicine which requires long acquisition times. We have developed a method for image correction between the respiratory-gated (RG) PET images in different respiration phases or breath-hold (BH) PET images in an inconsistent respiration phase. In the method, the RG or BH-PET images in different respiration phases are deformed under two criteria: similarity of the image intensity distribution and smoothness of the estimated motion vector field (MVF). However, only these criteria may cause unnatural motion estimation of lung. In this paper, assuming the use of a PET-CT scanner, we add another criterion that is the similarity for the motion direction estimated from inhalation and exhalation CT images. The proposed method was first applied to a numerical phantom XCAT with tumors and then applied to BH-PET image data for seven patients. The resultant tumor contrasts and the estimated motion vector fields were compared with those obtained by our previous method. Through those experiments we confirmed that the proposed method can provide an improved and more stable image quality for both RG and BH-PET images.

## 1. Introduction

Positron emission tomography (PET) is one of the useful modalities for tumor diagnosis of thoracoabdominal organs. Due to respiratory organ motion during image acquisition, however, images are affected motion blur. The respiratory-gated (RG) image acquisition technique can overcome this problem [1, 2]. If the projection data are collected in only a limited respiratory phase such as inspiration or expiration, less blurred images can be reconstructed from those data; however, because the detected counts are decreased by gating, a long acquisition time is required to accumulate sufficient counts. For example, in case that by gating only one-fourth period is used for data acquisition in each one respiratory cycle, four times longer acquisition time than the normal PET imaging is required in order to achieve the equivalent statistics.

One solution is to use all respiration phases by multiple gating, reconstruct the corresponding multiple images, correct the deformation between those images, and finally sum the corrected images. In this paper we call this the registration and summation method (RSM) and several articles have presented such an approach [3–8]. Rigid or affine transformations between two images are not sufficient for data where different organs inside the human thorax undergo different motions with varying directions and amplitudes. Nonrigid transformation is needed for such deformation correction. We have proposed a method for nonlinearly correcting the motion of the lung between RG reconstructed images in different respiratory phases and adding them together to obtain an image with less motion blur and less noise [3]. A similar method proposed by Dawood et al. [4, 5] utilizes a global optical flow algorithm for motion correction of images in individual gates.

As another imaging technique to avoid the respiratory motion blur, a breath-hold (BH) acquisition technique has recently been studied actively [9–13]. In this technique, a patient is asked to hold his/her breath for 10 to 30 s as the image acquisition is performed. Since one period is too short to accumulate enough radiation counts, this BH and acquisition are repeated. Summation of those obtained images provides a nonblurred and less granularity image with signal to noise ratio equivalent to the conventional PET image. In practice, however, a patient cannot hold his/her breath at the same timing of breathing. If the timing of the BH is not the same, the summed image still has blur. Thus, we proposed applying our image registration method developed for RG images to the BH images [14].

In our method, the RG or BH-PET images are deformed under two criteria: (1) similarity of the image intensity distribution and (2) smoothness of the estimated motion vector field (MVF). However, using only these criteria may cause unnatural motion estimation of lung when the image contrast is low and thus the texture information does not function for image registration. Nowadays, PET-CT scanners are getting more and more diffuse. CT images offer high contrast anatomical images and thus it is easier to obtain accurate motion information from inhalation and exhalation CT images.

In this paper, assuming the use of a PET-CT scanner, we added another criterion: the similarity to the motion direction estimated from two CT images in different respiration phases [15]. The proposed method was first applied to a numerical phantom XCAT [16] to confirm the basic idea and effectiveness. The results for the proposed method were compared with those by our previous method and a simple summation method in which the multiple images were just summed without any registration. The proposed method was also applied to clinical data composed of BH images at expiration and results were compared with those of the other two methods as well.

## 2. Materials and Methods

This method can be applied to a set of both RG and BH images. In the case of RG imaging, several PET images in different respiratory phases are obtained. In the case of BH imaging, PET images are acquired repeatedly in the same respiratory phase. Then, those images are deformed to match a reference image and all images are summed to get better image quality. Free form deformation is used in the registration step [17].

### 2.1. Previous Method

As mentioned above, in our previous RSM, PET images are deformed under two criteria: (1) similarity of the image intensity distribution and (2) smoothness of the estimated MVF. The deformation region is composed of a number of control points originally arranged in a matrix. The control points are defined in a floating image and moved so as to satisfy the criteria. Simulated annealing algorithm [18] is used for this optimization.

In the following formula, the pixel values of the reference image and the floating image are represented by *I*_ref_ and *I*_def_, respectively. The similarity of image intensity distribution at the location of the *i*th control point **x**_*i*_ is evaluated by

(1) $$\begin{array}{c} E_{1,i}={\left\{\;I_{ref}\left(\;x_{i}\;\right)-I_{def}\left(\;x_{i}+d_{i}\;\right)\;\right\}}^{2}, \end{array}$$

where **d**_*i*_ = (*u*_*i*_, *v*_*i*_, *w*_*i*_) denotes displacement of the *i*th control point in the floating image.

The second criterion is the smoothness of the estimated MVF for the floating image and evaluated at **x**_*i*_ by the Frobenius norm of a Hessian matrix using the next equation

(2) $$\begin{array}{c} E_{2,i}={\left\|\;H\left(\;u_{i}\;\right)\;\right\|}_{F}^{2}+{\left\|\;H\left(\;v_{i}\;\right)\;\right\|}_{F}^{2}+{\left\|\;H\left(\;w_{i}\;\right)\;\right\|}_{F}^{2}. \end{array}$$

Here *H*(*f*) represents a Hessian matrix, that is, a square matrix of second-order partial derivatives of scalar-valued function *f*, and ‖*H*(*f*)‖_*F*_^2^ represents its Frobenius norm. Frobenius norm is a square root of square sum of each element.

A combined criterion of the deformation over the image is given by

(3) $$\begin{array}{c} E_{total}=\sum_{i}\left(\;\alpha_{1}E_{1,i}+\alpha_{2}E_{2,i}\;\right), \end{array}$$

where *α*_1_ and *α*_2_ are weights to adjust the balance of the two terms. The summation is carried out over the image. To determine the values of these weights, *α*_1_ and *α*_2_, some perturbations are given to initial displacements and the resultant change in the value of each term of the combined criteria is evaluated. Then, the weights are adjusted so that the contribution of each term becomes similar.

### 2.2. Proposed Method

The proposed RSM consists of two steps. In step 1, an MVF is estimated from two CT images. In step 2, PET images are registered and summed making use of the estimated MVF. Details of each step are described below.

#### 2.2.1. Step  1: Estimation of MVF from CT Images

An MVF is estimated through the registration between inhalation and exhalation CT images. Free form deformation is applied to the CT image registration. In this registration, three criteria are used. The first two are similarity of the image intensity distribution and the smoothness of the MVF as used in the previous RSM.

The third criterion is the similarity for the motion vectors at neighboring feature points. Unlike PET images, marked corresponding feature points such as tracheobronchial bifurcation can manually be found from the two CT images. If we determine several feature points in a reference image and the corresponding points in a floating image, those correspondences should be maintained in the registration. The neighboring area of a feature point should have similar motion to that of the feature point. A measure of deformation taking into account the correspondence of control points at **x**_*i*_ is given by the following formula:

(4) $$\begin{array}{c} E_{3,i}=\overset{J}{\underset{j=1}{\sum }}\frac{\left\|d_{i}-d_{f,j}\right\|}{\left\|x_{i}-f_{j}\right\|}, \end{array}$$

where **f**_*j*_ represents the location of the *j*th feature point and **d**_*f*,*j*_ represents the motion vector of the *j*th feature point. The value *J* represents the total number of feature points used. If **x**_*i*_ = **f**_*j*_, we substitute unity in the denominator. This is a weighted sum of the norm of the differences between the motion vector at the current position and those at preselected feature points. Weights are given by the inverse of the distance between the current position and the feature point. This weight plays a role where the motion vector of the current position is similar to those of neighboring feature points. In this step, the total evaluation equation of the deformation is represented as

(5) $$\begin{array}{c} E_{total}=\sum_{i}\left(\;\alpha_{1}E_{1,i}+\alpha_{2}E_{2,i}+\alpha_{3}E_{3,i}\;\right). \end{array}$$

Each of the constant values, *α*_1_, *α*_2_, and *α*_3_, is set as mentioned in (3).

#### 2.2.2. Step  2: Registration and Summation of PET Images

BH-PET or RG-PET images are deformed and summed to obtain a high quality image. In our previous RSM, the similarity of the image intensity distribution and the smoothness of the MVF were used. Afterward, we add one more constraint. That is, the similarity for the motion direction estimated from two CT images. This term is given by the angle between the current motion vector and that estimated from CT images. The term is given by

(6) $$\begin{array}{c} E_{4,i}=\left|\;b_{i}\;\right|\left|\;cos^{-1}\frac{\left|a_{i}\cdot b_{i}\right|}{\left\|a_{i}\right\|\left\|b_{i}\right\|}\;\right|, \end{array}$$

where **a**_*i*_ is a motion vector candidate at the current position and **b**_*i*_ is a motion vector obtained in step 1. *E*_4,*i*_ is the angle between **a**_*i*_ and **b**_*i*_ weighted by the length of **b**_*i*_. It should be noted that we do not consider the similarity in magnitude of the motion vector. In this criterion, we roughly assume that any point in the lung moves on the straight line connecting its inhalation point and exhalation point. Thus the MVF is constrained in angle but not in magnitude. The higher the degree of matching is, the less the cost is. If the motion vector from CT images is large, such motion should be taken into account. The magnitude of **b**_*i*_ in (6) works for that purpose. If **a**_*i*_ = 0 or **b**_*i*_ = 0, *E*_4,*i*_ is set to 0. In this step, the evaluation equation of the deformation is represented as

(7) $$\begin{array}{c} E_{total}=\sum_{i}\left(\;\alpha_{1}E_{1,i}+\alpha_{2}E_{2,i}+\alpha_{4}E_{4,i}\;\right). \end{array}$$

**Figure 1:**
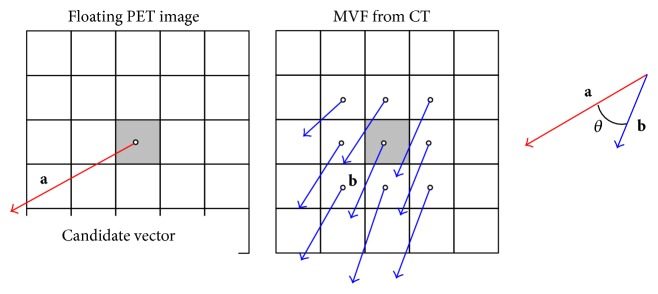
Similarity measurement of motion direction. A candidate motion vector is compared with the MVF estimated from the CT image.

Each of the constant values, *α*_1_, *α*_2_, and *α*_4_, is again set as mentioned in (3).  Figure 1 schematically shows the effect of the additional term.

## 3. Preliminary Tests

### 3.1. Numerical Phantom Test

We performed a test with a numerical phantom, XCAT, to verify the effectiveness of the proposed method. In this test, we assumed RG imaging. CT images used in step 1 and PET images used in step 2 were created from the XCAT phantom. In the simulation, CT images were acquired in the end-inhalation and end-exhalation. On the other hand, in PET imaging, the respiration period was divided into ten phases and images were acquired in each phase. For each PET image, Poisson noise whose level was similar to clinical data was added. Image sizes of CT and PET were 600 × 600 × 115 voxels and 102 × 102 × 58 voxels, respectively. Voxel sizes of CT and PET were 0.68 × 0.68 × 2.00 mm^3^ and 4 × 4 × 4 mm^3^, respectively.

**Figure 2:**
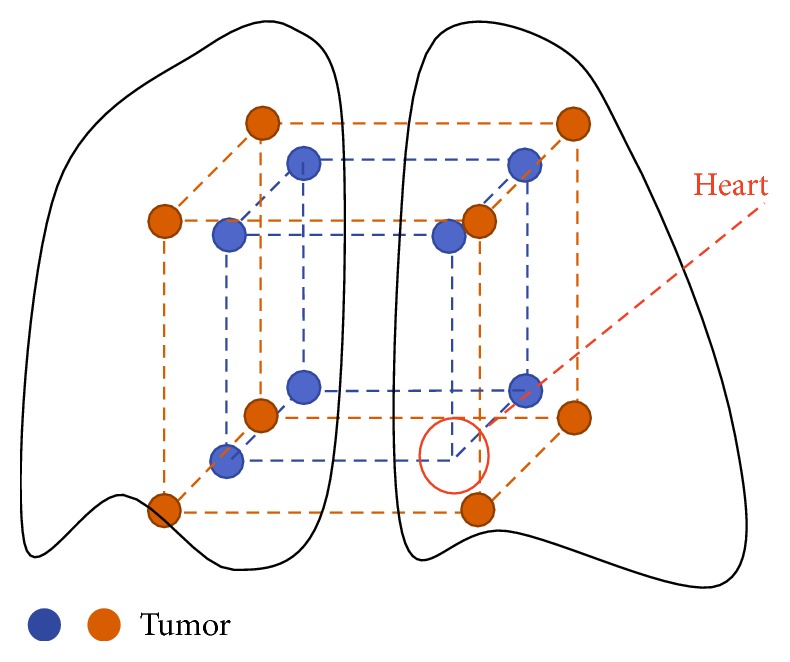
Location of 15 simulated small tumors in lung of XCAT phantom.

As feature points for getting the MVF of CT images, 13 bifurcation points were selected and used. Fifteen small, low-contrast tumors were located in the lung as shown in Figure 2. Those tumors corresponded to vertices of small and large cubes but one vertex located in the heart was omitted. In this study, the activity ratio of the tumor and background was given by 1.5 : 1. We set the concrete pixel values for tumors and the other regions in the lung as 24 and 36, respectively, for each respiratory phase image. Registration was carried out so that images in the second to tenth phases were matched to the first phase.

### 3.2. Clinical Data Measurements

We applied the previous and the proposed RSMs to seven sets of clinical FDG-PET images. The study has been approved by Yamaguchi University Hospital and all participant patients provided informed consent for the data collection. A PET-CT scanner, Gemini GXL 16, Philips Medical System, was used. ^18^F FDG (3.5 MBq/Kg) was administered to each patient and 60 min later the image acquisition was performed. The PET images were acquired under a BH imaging protocol which repeated a 10–15 s long BH six times. Such a BH protocol was performed at both exhalation and inhalation.

We performed an experiment using the clinical data. In the clinical experiment, we focused on the set of exhalation BH-PET images only. As mentioned earlier, even under the BH condition, there is variation in the respiration phase among BH images to some extent. We used these data sets to examine whether the proposed RSM was able to improve BH-PET image quality.

Table 1 shows the properties of the data used such as the number of CT slices, voxel size of CT, the number of feature points in the CT images used in the proposed RSM, image size of each PET slice, and the number of PET slices. As common properties, the image size of a CT slice was 512 × 512 pixels and the voxel size of PET was 4 × 4 × 4 mm^3^.

### 3.3. Quantitative Evaluations

Two kinds of quantitative evaluations were performed. The first one was about the pixel value itself or the contrast of the tumor. In the phantom data experiment, we evaluated the pixel values of the tumor itself because we can compare them with the ideal values. On the other hand, in the clinical data experiments, the contrast of the tumor to the background was used. This is defined by

(8) $$\begin{array}{c} R=\frac{\left(1/27\right)\sum_{i\in ROI}I_{i}}{\left(1/N\right)\sum_{i}I_{i}}, \end{array}$$

where *I*_*i*_ is the pixel value of the finally obtained PET image. ROI is defined by 3 × 3 × 3 = 27 voxels, the center of which corresponds to the peak value of the tumor region. *N* is the total number of voxels of the PET image. Namely, the value *R* was calculated by the ratio of the mean value in a small region centering on the tumor peak and the mean value of the whole image.

The second kind of evaluation used the angle between the finally estimated motion vector and that obtained from CT images.

## 4. Results and Discussion

### 4.1. Numerical Phantom Test

**Figure 3:**
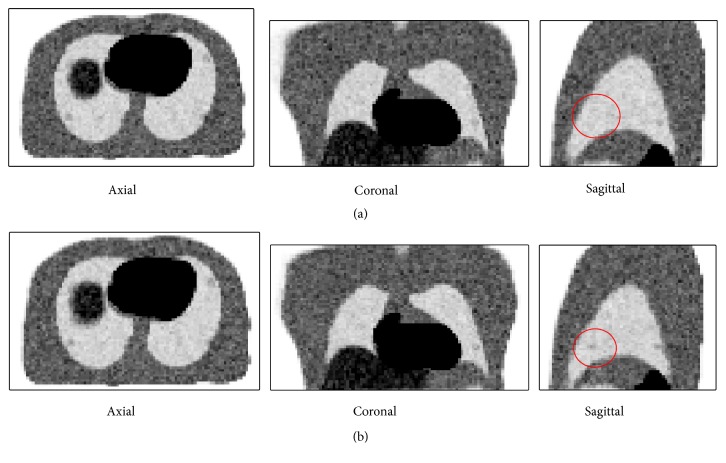
PET images obtained by RSMs. (a) Previous RSM. (b) Proposed RSM. In sagittal images, a ROI (region of Interest) is shown that indicates the presence of tumor (red circles).

Figure 3 shows the results of the numerical phantom test.  Figure 3(a) shows the resultant image by the previous RSM and Figure 3(b) shows that by the proposed RSM. No significant difference was observed between the images. However, in the lower left part in the sagittal image of the proposed RSM, a small tumor was visualized, while the previous RSM failed to visualize it.

Pixel values of 15 tumors were evaluated. For each tumor, 3 × 3 × 3 voxels in the tumor region were defined as the ROI and the mean values were evaluated.  Table 2 shows the mean and maximum value of the 15 values. Since the given value at a pixel in a tumor was 36 for each respiratory phase image, the ideal value in the tumor in the summed image was 360. In fact, interpolation processing degraded the ideal value slightly. The mean value obtained by the simple summation was the smallest. Both the previous RSM and the proposed RSM gave better values than the simple summation. In comparing results for the two RSMs, except for two tumors, the proposed RSM provided higher pixel values.

**Figure 4:**
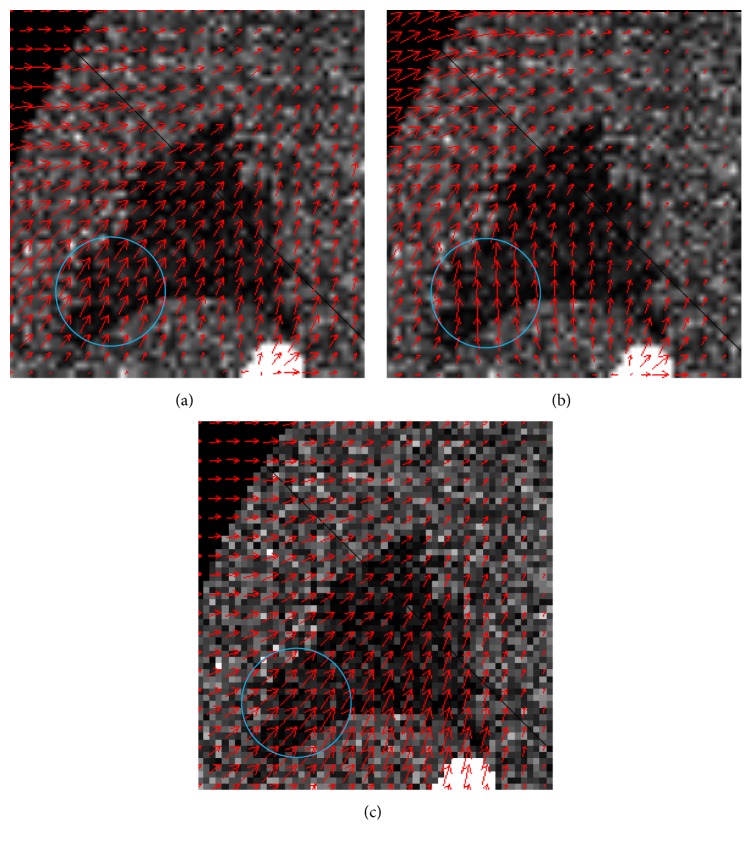
MVFs in the phantom data experiment. (a) MVF from CT images. (b) MVF by the previous RSM. (c) MVF by the proposed RSM.

MVFs were also compared.  Figure 4 shows the MVFs of the XCAT phantom estimated (a) from two CT images, (b) by the previous RSM and (c) by the proposed RSM. Here the motion vector was calculated at each grid point and displayed as a red arrow. Since the grid points were arranged so as to cover the whole cubic region of the CT and PET volume images, the grid points outside the body were also estimated. Those vectors were ignored in the evaluation. While the MVF estimated by the previous RSM was inconsistent with that from the CT images, the MVF by the proposed RSM was consistent with that from the CT images. The difference was especially remarkable within the blue circle.

We evaluated the similarity of angles between the estimated motion vectors and those from CT images.  Table 3 shows these results. Here two estimated motion vectors were projected onto axial, coronal, and sagittal planes and the angles between the two vectors in each plane were averaged over the image for each respiratory phase. It was clear that there were big errors in the results obtained by the previous RSM.

### 4.2. Clinical Data Evaluations

**Figure 5:**
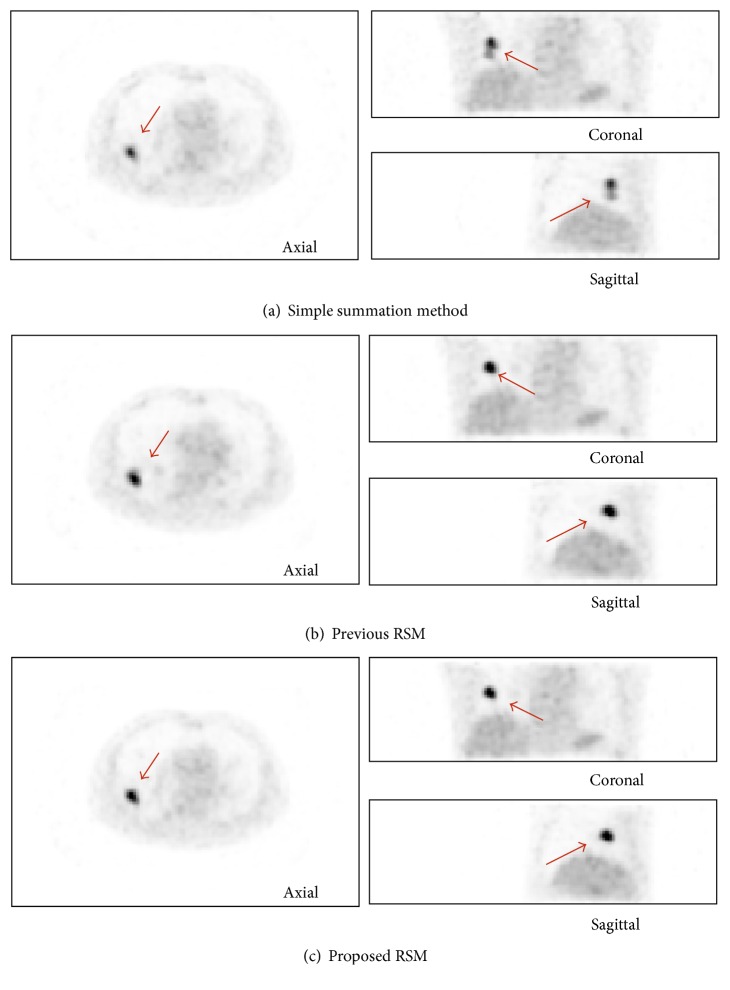
An example of results of PET image registration and summation. Only exhalation images were used. (a) Simple summation. (b) Previous RSM. (c) Proposed RSM.

Table 4 summarizes the values of contrast defined by (8). Here the simple summation, the previous RSM, and the proposed RSM were compared. In many cases, no marked increase of the value was observed. For patient number 6 the proposed RSM clearly achieved higher contrast. The corresponding images are shown in Figure 5. (a), (b), and (c) correspond to the simple summation, the previous RSM, and the proposed RSM, respectively. In this example, we supposed that the patient could not repeat the same breath-hold and thus the tumor position had changed. The simple summation method produced a blurred tumor. In the previous RSM and the proposed RSM the blur was successfully reduced.

**Figure 6:**
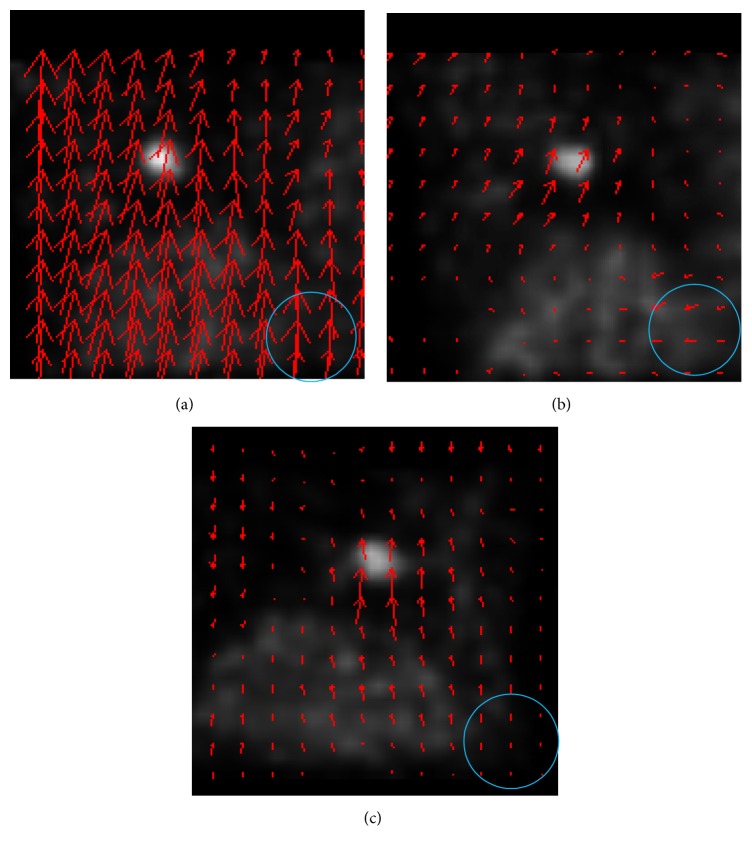
MVF obtained using only exhalation images. (a) From CT images. (b) Previous RSM. (c) Proposed RSM.

MVFs in these clinical data were compared as well.  Figure 6 shows an example of MVFs. The arrangement of the figures is the same as in Figure 4. While the MVF by the previous RSM was different from that obtained from CT images, the MVF by the proposed RSM was similar to that obtained from CT images although the length itself was not similar.

The angles of the motion vector estimated by the RSMs were compared with those estimated from CT images.  Table 5 shows these results. As presented in Table 3, the angles between two vectors in three orthogonal planes were evaluated.  Table 5 clearly shows that the previous RSM estimated the motion vectors with significant differences compared to the motion vectors from the CT images.

In many cases, the apparent image quality of the PET image obtained by the previous RSM was similar to the simple summation and the proposed RSM. However, as seen in Table 5, the motion vectors by the previous RSM were different from those from CT images. Since the image similarity was dominant in the previous RSM, the tumor regions with high contrast tended to gather together. However it should be noted that such a summation might lead to incorrect excessive enhancement.

## 5. Conclusion

In this paper, by assuming use of a PET-CT scanner, we added a similarity measure on the motion direction estimated from two CT images in different respiration phases as another criterion for the RSM. The proposed RSM was applied to a numerical phantom and clinical BH-PET images and compared with our previous RSM which used only the similarity of the image intensity distribution and the smoothness of MVF as optimization criteria. Through results we confirmed that the proposed RSM can achieve better and more stable image quality for both RG and BH-PET images. As a future task, the proposed RSM should be applied to real RG-PET images to confirm its practical effectiveness.

## Figures and Tables

**Table 1:** CT and PET image data used in the experiment. For all patient data, image size of CT was 512 × 512 pixels and the voxel size of PET image was 4 × 4 × 4 mm^3^.

Patient ID number	CT		PET		
	Slices	Voxel size (mm^3^)	Feature points	Image size (pixels)	Slices
1	38	1.17 × 1.17 × 5	6	150 × 150	48
2	91	0.68 × 0.68 × 2	8	87 × 87	46
3	88	0.68 × 0.68 × 2	10	87 × 87	44
4	91	0.68 × 0.68 × 2	12	87 × 87	46
5	91	0.68 × 0.68 × 2	5	87 × 87	46
6	36	1.17 × 1.17 × 5	7	150 × 150	45
7	93	0.68 × 0.68 × 2	23	87 × 87	46

**Table 2:** Mean and maximum pixel values of tumors by simple summation, previous RSM, proposed RSM, and ideal case.

	Simple summation	Previous RSM	Proposed RSM	Ideal
Mean	284.9	321.7	329.5	353.4
Maximum	304.2	351.7	358.5	371.6

**Table 3:** Evaluation of estimated motion angle in phantom experiment.

Respi. phase	Previous RSM [degree]			Proposed RSM [degree]		
	Axial	Coronal	Sagittal	Axial	Coronal	Sagittal
2	13.0	6.9	6.6	1.7	2.2	2.5
3	12.7	6.3	7.0	2.3	2.2	1.9
4	17.1	10.2	9.6	2.1	2.0	2.3
5	18.0	8.7	11.7	2.7	1.7	1.7
6	16.5	8.3	10.1	2.5	2.2	2.5
7	18.0	9.3	10.2	2.4	2.3	2.3
8	14.6	8.3	9.1	1.9	2.2	2.4
9	13.3	7.4	6.1	2.0	2.6	2.2
10	11.5	6.1	5.7	2.2	2.6	1.9

**Table 4:** Evaluation of contrast of tumor in clinical data experiment.

Patient ID number	Simple sum	Previous RSM	Proposed RSM
1	20.0	21.0	21.5
2	12.6	11.6	11.6
3	30.0	32.5	32.5
4	6.4	6.6	6.5
5	24.3	24.6	24.7
6	**42.6**	**48.1**	**52.1**
7	23.9	26.9	27.0

**Table 5:** Evaluation of estimated motion angle in the clinical data experiment.

Patient ID number	Previous [degree]			Proposed [degree]		
	Axial	Coronal	Sagittal	Axial	Coronal	Sagittal
1	27.7	24.3	18.5	3.0	2.9	2.6
2	16.3	12.4	11.5	2.7	2.3	3.0
3	13.1	13.0	15.0	3.1	4.2	2.6
4	16.3	9.3	19.5	3.0	2.7	3.7
5	17.1	10.4	8.7	6.5	3.3	3.1
6	17.7	17.4	11.3	3.0	3.3	3.2
7	26.8	16.3	15.8	4.6	2.3	3.1
